# Physical therapy students’ perceptions of the educational environment at physical therapy institutes in Pakistan

**DOI:** 10.3352/jeehp.2020.17.7

**Published:** 2020-02-24

**Authors:** Muhammad Adeel, Asad Chaudhry

**Affiliations:** 1 College of Biomedical Engineering, Taipei Medical University, Taipei, Taiwan; 2 District Headquarter Hospital, Narowal, Pakistan; Hallym University, Korea

**Keywords:** Atmosphere, Learning, Pakistan, Physical therapy modalities, Students

## Abstract

This study assessed doctor of physical therapy (DPT) students’ perceptions of the educational environment at public and private physical therapy institutes in Pakistan. This cross-sectional study was conducted at 6 physical therapy institutions in Punjab, Pakistan from April 2018 to December 2019. In total, 500 Dundee Ready Educational Environment Measure (DREEM) questionnaires were distributed among DPT students identified through convenience sampling (response rate, 86.4%). The correlations between each item of the DREEM score were analyzed. The mean overall DREEM score was 128±19.63 for all 5 subscales (range, 33 to 166; standard error of the mean, 0.954). The correlations of atmosphere, learning, and self-perception with the overall educational environment were r=0.896, r=0.853, and r=0.846, respectively. Student-centered approaches were found to be more effective than teacher-centered approaches for promoting a positive educational environment.

## Background/rationale

The doctor of physical therapy (DPT) degree is received through a 5-year educational program consisting of courses on basic medical sciences in the first 2 years and clinical sciences in next 3 years. All students enrolled in DPT programs at 6 public and private medical institutions in Punjab, Pakistan were eligible for participation in this study. In Pakistan, the DPT curriculum is supervised by the Higher Education Commission, and is categorized as a 5-year degree/10-semester program with 175 credit hours; after completing the degree, a 1-year residency is required [1]. Ultimately, what a student perceives in his/her learning environment will be reflected in his/her professional life. Therefore, it is important to obtain frequent feedback in order to make adjustments, correct errors, and maintain momentum. Students’ expectations regarding the educational environment can be a basis for making improvements that enhance the educational environment. Significant learning is favorably correlated with insights into students’ educational environment, which influence students’ learning outcomes by shaping the way students learn, as well as why and what they learn [2,3]. However, most previous researchers concluded that the overall educational environment ranges from average to poor across the globe. No research has been conducted exclusively among DPT students in Pakistan.

## Objectives

The aim of this study was to evaluate DPT students’ perceptions of their educational environment to help address fluctuations in the current typical educational environment according to students’ needs.

## Ethics statement

The study received approval from the research and ethics committee of the School of Physiotherapy, King Edward Medical University, Lahore (IRB-SPT-0004562). Informed consent was obtained from each participant before data collection.

## Study design

This cross-sectional study was conducted at 6 public and private medical institutions in Punjab from April 2018 to December 2019. Data were collected using a demographic survey and the Dundee Ready Educational Environment Measure (DREEM) questionnaire [4].

## Materials and/or subjects

In total, 500 (response rate, 86.4%) printed questionnaires were distributed among DPT students identified through convenience sampling at 2 public (King Edward Medical University, School of Physiotherapy and Children Hospital Lahore, School of Allied Health Sciences) and 4 private (Imperial College of Business Studies Department of Physical Therapy & Rehabilitation Sciences, University of Sargodha Lahore Campus Department of Physical Therapy, University of Lahore University Institute of Physical Therapy, and Federal Institute of Health Sciences Department of Physical Therapy) physical therapy institutions. Questionnaires were distributed among students after their scheduled classes. Use of the questionnaires was permitted by the original author of the tool. An administrative member of each institution helped in the data collection process and survey completion.

The DREEM scale is most reliable, valid, and culture-nonspecific public tool that is used internationally to assess students’ perceptions of their educational environment. It consists of 50 questions with 5 subscales: (1) students’ perceptions of learning (SPL), (2) students’ perceptions of teaching (SPT), (3) students’ academic self-perceptions (SASP), (4) students’ perceptions of atmosphere (SPA), and (5) students’ social self-perceptions (SSSP).

Each question is rated on a 5-point Likert scale (0, strongly disagree; 1, disagree; 2, unsure; 3, agree; 4, strongly agree). This inventory has a total score of 200, which represents the ideal learning atmosphere as perceived by students. The lowest possible rating is a score of 0. The subscale scores can be evaluated against generic parameters, and individual question ratings can also be reviewed to pinpoint specific advantages and disadvantages [5].

## Statistical analysis

IBM SPSS ver. 21.0 (IBM Corp., Armonk, NY, USA) was used for the statistical analysis. The demographic variables are presented as number and percentage, while the DREEM variables, total DREEM scores, and scores on the 5 subscales are presented as mean values with standard deviations. The level of statistical significance was P<0.05. The correlations of scores for all DREEM items were analyzed.

## Key results

The study presented data from 423 participants (77 participants did not respond) with a mean age of 22.95±2.413 years, of whom 47.5% (n=201) were male and 52.5% (n=222) female. The questionnaires were completed by students at 2 public (26.2%, n=111) and 4 private (73.8%, n=312) institutes. Programs with an annual examination system accounted for 48.2% (n=204) of responses, while those with a semester system accounted for 51.8% (n=219) (Table 1). Raw data were available from Dataset 1.

The overall DREEM score was 128±19.63 for all 5 subscales (range, 33 to 166; standard error of the mean, 0.954). The Cronbach α for the DREEM scale was 0.806. The descriptive statistics of each subscale and inter-class correlations are shown in Tables 2 and 3, respectively.

The reliability of the DREEM scale was 80.6%. It was enhanced by removing the following items: “The teaching overemphasizes factual learning,” “The teaching is too teacher centered,” “My problem solving skills are being developed well here,” and “I am rarely bored with this course.” The subscales of SASP and SPA exhibited good reliability, with Cronbach α values of 0.749 and 0.734, respectively. The highest mean values were found for SPL and SPA, at 31.99±5.24 and 31.20±6.42, respectively (Table 2).

Perceptions of atmosphere, learning, and self-perception were strongly linked with the overall educational environment at physical therapy institutes (r=0.896, r=0.853, and r=0.846 respectively). The correlations of SPT and SSSP with the overall DREEM score were lower, but still meaningful (r=0.728 and r=0.696 respectively). Out of these 5 subscales, the highest correlation was found between SPL and SASP (r=0.708) (Table 3).

Interpretation and suggestions: Evaluating the educational environment is an important method for ensuring quality and distinguishing dimensions of progress. The academic atmosphere not only helps account for students’ fulfillment, but also influences students’ conduct and predicts their achievements. The educational environment can best be measured in terms of the total and subscale scores of the DREEM questionnaire [6]. In this study, student-centered perceptions in terms SASP, SPA, and SPL showed good reliability (α=0.749, 0.734, and 0.684, respectively) and mean scores (22.75±4.72, 31.20±6.42, and 31.99±5.24, respectively), suggesting that students had more favorable perceptions of the learning environment. The SSSP domain was found to need further strengthening, as shown by the finding that it had the lowest reliability and mean scores (α=0.347 and 17.37±3.36, respectively). Such improvements are already being implemented through a holistic system that provides mentoring, academic, and psychological health support and advice [6]. This study highlights that teacher-centered approaches influence the educational environment less favorably than student-centered approaches, as shown by low reliability (α=0.575) and mean scores 25.64±4.24 of the SPT subscale and the finding that the Cronbach α score for the SPL subscale was improved by removing the “teacher overemphasizes factual learning” item.

## Comparison with previous studies

In a study conducted by Rehman et al. [7] in 2016, the mean total score on the DREEM was 126/200, which was higher or comparable to those reported by other studies from medical colleges around the world. Based on that score, the bachelor’s medical education program of Aga Khan University in Karachi, Pakistan was categorized as more positive than negative [7]. Research conducted at an Australian university highlighted that students reported overall positive perceptions of the educational environment, with a high DREEM score of 137.3±18.3. This reflected a student-centered approach at the university, which caused the students to show a positive outcome [8].

A previously published paper suggested that the highest-rated items were knowledgeable teachers, good friends, and confidence to pass the exams, although 3 of the most troublesome elements were the use of an unsuitable method for a stressful situation, failure to remember everything, and a focus on factual learning [9]. The researchers concluded that stress, tiredness, and insufficient feedback from teachers provide a stimulus for negative student perceptions, while the domains that influence positivity and a feeling of happiness in students were social comforts, school friends, and accommodations [10].

## Strengths and limitations

This survey had a good response rate and consistency. Further study is needed to analyze the educational environment for specific years in the program. The generalizability of the findings is limited because this study included only 6 institutes from a single province (Punjab).

## Conclusion

In conclusion, student’s academic self-perceptions, atmosphere, and learning perceptions affected the educational environment at the physical therapy institutes analyzed in this study. Student-centered approaches were found to be more effective for promoting a positive educational environment than teacher-centered approaches. This study may yield insights into ways of modifying teacher-oriented behaviors to improve academic performance by focusing on ways of teaching and students’ social life.

## Figures and Tables

**Table 1.** Characteristics of participants (n=423)

Characteristic	Value
Gender	
Male	201 (47.5)
Female	222 (52.5)
Institute type	
Government	111 (26.2)
Private	312 (73.8)
Exam system of the institute	
Annual	204 (48.2)
Semester	219 (51.8)

Values are presented as total number of (%).

**Table 2.** Descriptive statistics of DREEM scores

	Mean±standard deviation	Subscale score interpretation	α
Students’ perceptions of learning	31.99±5.24	A more positive approach	0.684^a)^, b^b)^
Students’ perceptions of teachers	25.64±4.24	Moving in the right direction	0.575
Students’ academic self-perceptions	22.75±4.72	Feeling more on the positive side	0.749^c)^
Students’ perceptions of atmosphere	31.20±6.42	A more positive atmosphere	0.734
Students’ social self-perceptions	17.37±3.36	Not too bad	0.347^d)^
Total DREEM score	128.96±19.63	More positive than negative	0.806

DREEM, Dundee Ready Educational Environment Measure.

a) Improved to 0.746 if the “teaching overemphasizes factual learning” item was removed.

b) Improved to 0.754 if the “teaching is too teacher-centered” item was removed.

c) Decreased to 0.705 if the “my problem solving skills are being developed well here” item was removed.

d) Improved to 0.419 if the “I am rarely bored with this course” item was removed.

**Table 3.** Correlations between scores of DREEM items

	SPL	SPT	SASP	SPA	SSSP
SPT	0.497^**^				
SASP	0.708^**^	0.478^**^			
SPA	0.690^**^	0.592^**^	0.671^**^		
SSSP	0.487^**^	0.419^**^	0.541^**^	0.530^**^	
DREEM total score	0.853^**^	0.728^**^	0.846^**^	0.896^**^	0.696^**^

DREEM, Dundee Ready Educational Environment Measure; SPL, students’ perceptions of learning; SPT, students’ perceptions of teaching; SASP, students’ academic self-perceptions; SPA, students’ perceptions of atmosphere; SSSP, students’ social self-perceptions.

** P<0.01; Correlation was significant at the 0.01 level (2-tailed).

## Footnotes

**Data availability** Data files are available from https://doi.org/10.7910/DVN/IJXZWZ Dataset 1. Raw data of the responses by subjects.

## Supplementary materials

Data files are available from https://doi.org/10.7910/DVN/IJXZWZ

Supplement 1. Audio recording of the abstract.
